# The Best Services Trial (BeST?): a cluster randomised controlled trial comparing the clinical and cost-effectiveness of New Orleans Intervention Model with services as usual (SAU) for infants and young children entering care

**DOI:** 10.1186/s13063-022-06007-3

**Published:** 2022-02-07

**Authors:** Karen Crawford, Bridie Fitzpatick, Lynn McMahon, Matt Forde, Susanne Miller, Alex McConnachie, Martina Messow, Marion Henderson, Emma McIntosh, Kathleen Boyd, Dennis Ougrin, Phil Wilson, Nicholas Watson, Helen Minnis

**Affiliations:** 1 Mental Health and Wellbeing, Institute of Health and Wellbeing, University of Glasgow, Glasgow, UK; 2 Centre for General Practice and Primary Care, University of Glasgow, Glasgow, UK; 3 Stratified Medicine Scotland Innovation Centre, University of Glasgow, Glasgow, UK; 4 NSPCC, London, UK; 5 Glasgow Health and Social Care Partnership, Glasgow, UK; 6 Robertson Centre for Biostatistics, University of Glasgow, Glasgow, UK; 7 School of Social Work and Social Policy, University of Strathclyde, Glasgow, UK; 8 Health Economics and Health Technology Assessment, University of Glasgow, Glasgow, UK; 9 Institute of Psychiatry, Psychology and Neurodevelopment, King’s College London, London, UK; 10 Centre for Rural Health, University of Aberdeen, Aberdeen, UK; 11 Centre for Disability Research, University of Glasgow, Glasgow, UK

**Keywords:** Infant mental health, Cluster randomised controlled trial (RCT), Social care, Foster care, Health economics, Judiciary, Parenting capacity, Adoption, Vulnerable families

## Abstract

**Background:**

Abused and neglected children are at increased risk of health problems throughout life, but negative effects may be ameliorated by nurturing family care. It is not known whether it is better to place these children permanently with substitute (foster or adoptive) families or to attempt to reform their birth families.

Previously, we conducted a feasibility randomised controlled trial (RCT) of the New Orleans Intervention Model (NIM) for children aged 0–60 months coming into foster care in Glasgow. NIM is delivered by a multidisciplinary health and social care team and offers families, whose child has been taken into foster care, a structured assessment of family relationships followed by a trial of treatment aiming to improve family functioning. A recommendation is then made for the child to return home or for adoption.

In the feasibility RCT, families were willing to be randomised to NIM or optimised social work services as usual and equipoise was maintained. Here we present the protocol of a substantive RCT of NIM including a new London site.

**Methods:**

The study is a multi-site, pragmatic, single-blind, parallel group, cluster randomised controlled superiority trial with an allocation ratio of 1:1. We plan to recruit approximately 390 families across the sites, including those recruited in our feasibility RCT. They will be randomly allocated to NIM or optimised services as usual and followed up to 2.5 years post-randomisation. The principal outcome measure will be child mental health, and secondary outcomes will be child quality of life, the time taken for the child to be placed in permanent care (rehabilitation home or adoption) and the quality of the relationship with the primary caregiver.

**Discussion:**

The study is novel in that infant mental health professionals rarely have a role in judicial decisions about children’s care placements, and RCTs are rare in the judicial context. The trial will allow us to determine whether NIM is clinically and cost-effective in the UK and findings may have important implications for the use of mental health assessment and treatment as part of the decision-making about children in the care system.

## Administrative information

Note: the numbers in curly brackets in this protocol refer to SPIRIT checklist item numbers. The order of the items has been modified to group similar items (see http://www.equator-network.org/reporting-guidelines/spirit-2013-statement-defining-standard-protocol-items-for-clinical-trials/).

**Table:** 

Title {1}	The Best Services Trial (BeST?): a cluster randomised controlled trial comparing the clinical and cost-effectiveness of New Orleans Intervention Model with Services as Usual (SAU) for infants and young children entering care
Trial registration {2a and 2b}.	Registered in ClinicalTrials.gov: NCT02653716 Effectiveness and Cost-effectiveness of the New Orleans Intervention Model for Infant Mental Health - Full Text View - ClinicalTrials.gov
Protocol version {3}	Version 7.0 12.05.2020
Funding {4}	NIHR Chief Scientist’s Office NSPCC
Author details {5a}	SPIRIT guidance: Affiliations of protocol contributors. Karen Crawford, Helen Minnis - Mental Health and Wellbeing, Institute of Health and Wellbeing, University of Glasgow Alex McConnachie and Martina Messow - Robertson Centre for Biostatistics, University of Glasgow Emma McIntosh and Kathleen Boyd, Health Economics and Health Technology Assessment, University of Glasgow Marion Henderson - School of Social Work and Social Policy, University of Strathclyde Phil Wilson – Centre for Rural Health, University of Aberdeen Matt Forde - NSPCC Susanne Millar - Glasgow Health and Social Care Partnership Bridie Fitzpatrick, Centre for General Practice and Primary Care, University of Glasgow Lynn McMahon, Stratified Medicine Scotland Innovation Centre, University of Glasgow Dennis Ougrin, Institute of Psychiatry, Psychology and Neurodevelopment, Kings College London
Name and contact information for the trial sponsor {5b}	SPIRIT guidance: Name and contact information for the trial sponsor. Emma McDonough, Sponsor’s Representative, NHS Greater Glasgow and Clyde Research and Innovation Ward 11 Dykebar Hospital Grahamston Road Paisley PA2 7DE Email: Emma.McDonough@ggc.scot.nhs.uk Tel: 0141 314 4011
Role of sponsor {5b}	Sponsor has delegated responsibility for the conduct of the trial. The sponsor’s responsibility is to confirm there are proper arrangements in place to initiate, manage, monitor and finance the study and to ensure proper indemnity is in place.

## Introduction

### Background and rationale {6a}

Children who experience maltreatment in early life are at increased risk of a range of adverse mental and physical health outcomes including substance misuse, cardiovascular disease [1], self-harm, suicide attempts and suicide [2–5]. Early childhood adversity and associated disorders impose a massive financial burden on individuals, families and society [6]. Maltreated young people are overrepresented in inpatient psychiatric units, although they can benefit from intensive community care services [7]. Maltreatment-associated mental health problems, e.g. conduct disorder and attention deficit hyperactivity disorder (ADHD), are treatable [8, 9], so improving the mental health of young maltreated children is likely to yield substantial rewards in terms of the health and productivity of individuals, families and the population as a whole [10].

For maltreated children, the most important intervention may be the provision of a safer and more nurturing home environment: research on sensitive periods in neural development suggests that addressing inadequate care in the early months and years of life may improve neural circuits underpinning social development [11] and allow maltreated children to reach their full developmental potential [12]. More than 57,000 children were in foster care in England in March 2020 and, in June 2019, 4800 children were in foster care in Scotland [13]—around 4 to 5 in every 1000 children [14, 15]. Recovery from the effects of early maltreatment can be rapid if safe nurturing care is achieved early enough, ideally in the first year of life [16], whereas robust predictors of poor outcome for maltreated children include prolonged pre-care exposure to multiple adversities, placement instability and “drift” in care [17]. Despite this knowledge, nearly a third of infants (31%) and nearly a quarter of children aged 1–4 years (24.5%) will return to foster care within 5 years of being reunited with their parents [18]. There is much current debate about the ethics of permanent care (i.e. adoption) for maltreated children [19, 20] and the timescales involved in making these decisions [21]. In the UK, adoption does not take place on average until at least 3 years of age [21] despite the presence of adversity in most cases since birth. There are currently no evidence-based interventions aiding social work services and the legal profession to make the difficult decision about whether a pre-school child in care should be adopted or returned home.

Because of the poor health outcomes for maltreated infants in our current system, new technologies should be tested that have the potential to provide safe, nurturing care in timescales that allow benefits in terms of optimal brain development. The most effective way to improve the mental health of young children is to target *both* child behaviour problems *and* parent-child relationships, and medium to large effect sizes on a range of outcomes have been noted with these strategies [22, 23]. Although there is evidence that specialised foster care can be cost-effective for young children requiring substitute care [24], there is a lack of focus on placement outcomes [23] and we are not aware of any previous or current randomised controlled trials (RCTs) addressing decisions about whether children should be adopted or returned home. The aim of the Best Services Trial is to provide evidence to inform future service developments and policy decisions about the most effective and cost-effective approach for assessment of and intervention for children entering foster care due to abuse and neglect. Our primary outcome is child mental health. We hypothesise that introducing the New Orleans Intervention Model (NIM) into care proceedings for maltreated pre-school children coming into care will be a clinical and cost-effective way of improving their mental health.

## Feasibility trial

A detailed feasibility trial (NCT01485510), funded by the Chief Scientist Office (CSO) for Scotland (December 2011–May 2015) then by UK children’s charity NSPCC (nspcc.org.uk) (June 2015 to December 2015), was conducted to determine whether a definitive multicentre UK RCT was feasible, acceptable and necessary, what the required size of a definitive RCT would need to be and to ascertain the optimal outcome measures for a definitive trial. The funding for the definitive trial commenced in January 2016, and a protocol for the definitive trial was published in 2017 [25], although methodological challenges experienced in the London site meant that the multi-site definitive trial did not begin until Autumn of 2017. Due to this delay, and then further delays due to COVID-19, a funding extension was sought and granted. Once it became clear, in early 2021, that the definitive trial would be achievable, a more detailed protocol paper was warranted to describe the various challenges and contextual changes that necessitated the methodological variations we describe below.

The timelines of the feasibility and definitive trials are as follows in Table 1.

**Table 1:** Timelines of stages of Best Services Trial

Stage	Feasibility RCT CSO funded	Bridging period NSPCC funded	NIHR funding/legal challenge to London randomisation	Definitive RCT with London site	End of recruitment	New end date with 2.5-year follow-up
Time	Dec 2011–May 2015	June 2015–Dec 2015	Jan 2015–28.08.2017	29.08.2017	31.7.21	29.02.2024
Randomisation condition	Consent > baseline > randomisation	Randomisation > consent > baseline		Consent > randomisation > baseline		
Key challenges	Very high recruitment rate and delays to accessing services	Waiting lists due to teams treating non-RCT families		Potential for bias to be introduced through post-randomisation baseline assessment—necessary to comply with judicial requirements		

These have included the following:

- A very high initial response rate during the feasibility trial caused us to carefully examine the ethics of consent in the target population. In order to try to improve the ethical basis for the study, we introduced randomisation before consent (modified Zelen randomisation [26]). Unanticipated consequences of randomisation before consent included delays in service delivery since the infant mental health teams, which had had their capacity modelled on likely trial recruitment numbers, became overwhelmed with treating non-trial participants who were randomised but did not then consent to the study.
- A judicial challenge to randomisation in London required us to carefully examine randomisation procedures to ensure they complied with both the NHS governance framework and the judicial framework. Although our randomisation-before-consent procedure had been instituted in an attempt to improve the ethical basis for the study, we realised that, on balance, it had raised additional ethical problems: families randomised to NIM were having to wait for the intervention for study-related reasons which was potentially detrimental [12]. Because of the very short timescales available for parenting capacity assessments imposed on English care proceedings by the English Family Justice Review [27], it was crucial that interventions could begin immediately after the child was placed in care, so we instituted a new system of randomisation after consent, but prior to baseline assessment.
- A reduction in numbers of children coming into foster care and a parallel increase in the use of kinship care placements in Glasgow led us to include children coming into an episode of kinship care where the local authority placed statutory governance around the placement. Permanence procedures for kinship placements had been amended, and it was determined that there would be sufficient oversight of such placements for them to have the same stability and safety as foster care placements and thus would be appropriate to be randomised to receive NIM or Case Management.

## Objectives {7}

The main objective of the trial is to establish if NIM is more effective in improving the mental health of maltreated infants and young children than services as usual (SAU), i.e. social work Case Management (CM) [28].

There are 3 secondary objectives: compared to social work Case Management SAU, to establish whether NIM is effective in improving the relationship between maltreated infants and young children and their primary caregiver, whether NIM effects more timely permanent placement decisions for maltreated children at 2.5 years post-randomisation and whether NIM is cost-effective in terms of the short-term mental health of the child and in terms of a long-term population health model.

## Trial design {8}

A multi-site, pragmatic, single-blind, parallel group, cluster randomised controlled superiority trial with an allocation ratio of 1:1.

## Methods: participants, interventions and outcomes

### Study setting {9}

The study settings are social services in the local authorities feeding into the two trial sites: Greater Glasgow and Clyde (Scotland) comprising Glasgow City Council and, from October 2018, Renfrewshire Council; and London (England), including 6 boroughs Croydon, Tower Hamlets, Sutton, Bromley, Newham and Barking and Dagenham. Although each site is in the UK, the social services and legal contexts in each differ considerably.

### Eligibility criteria {10}

#### Inclusion

Families are eligible for the trial if they have a child aged 0 to 60 months who enters care in the recruiting sites for reasons associated with maltreatment, during the study recruitment period. Participants are included in the trial upon completion of written informed consent from the birth family and the child’s foster or kinship family.

In the event of a subsequent child being taken into care among participating families, consent will be sought from parent(s) and carers to include the new child. If consented, the new child will not be randomised, but will instead be allocated to the same arm of trial as their previous family members. This is partly to avoid contamination, but also because the aim of NIM is to help the parent(s) become more nurturing for both the current and any future children, and if a parent has already had NIM, it might be confusing for them to receive an intervention with a different and potentially contradictory ethos.

#### Exclusion

Families will be excluded from the trial if the parent(s) is unavailable to take part in an intervention (for example, because of death, unknown whereabouts or long-term imprisonment).

If a family has been randomised previously but became ineligible without being exposed to either of the study interventions, then the family may be randomised again, should they become eligible at a later date.

### Who will take informed consent? {26a}

Trial recruitment coordinators (experienced social workers trained in conducting consent interviews) will be notified of a newly eligible (or potentially eligible) family via social workers or designated members of the legal team in each local authority area. They will confirm that birth families have received information sheets and videos about the trial and have assented to be approached by a recruitment coordinator. If so, we will arrange a meeting to discuss trial participation, answer any questions and, if proceeding, go through the informed written consent process. Subsequent meetings can be arranged if birth parents would like more time to decide about participation. Consent can be taken remotely in extenuating circumstances.

If parents agree to participate in the study, foster/kinship carers will be contacted by one of the research team to discuss their participation in the study and take consent.

### Additional consent provisions for collection and use of participant data and biological specimens {26b}

We take consent to access routinely collected health and social work data about the child and parent. Any specific requests for routine data from central sources (e.g. NHS or legal system routine data) will require a separate application and be governed under information governance legislation by the relevant bodies. The data will be used to monitor safety throughout the trial, to examine the representativeness of the trial sample, to augment the planned health economics analysis and to monitor current placements of children in the study to enhance retention rates at follow-up.

Data contributing to our time to permanent placement secondary outcome measure are gathered, blind to arm of trial, from social work and legal system records. These data include dates and recommendations of the parenting capacity assessment received, dates and recommendations made at permanence hearings, placement details and legal orders after permanence decisions were made.

In addition, routine data will be used as part of the process evaluation, for example to examine how the social care and legal context is related to children’s journeys through placement and to their mental health. This is described in a separate process evaluation protocol paper [29].

## Interventions

### Explanation for the choice of comparators {6b}

Families not randomised to NIM will receive services as usual (SAU), i.e. the assessment and intervention that social services and others normally implement when children are removed from parental care. SAU includes regular contact with families by a social worker Case Manager, assessment of family relationships and signposting of families towards existing services. Case Management was described and found to be effective in a randomised controlled trial conducted in the USA [28]. It is important to note that the Case Management evaluated in that study was similar to the minimum service routinely offered by social work departments across the UK, since “local authorities have a duty to safeguard…children … and…[provide] a range and level of services appropriate to the children’s needs” [30, 31]. Such Case Management services are not routinely available in the USA [32]. In Scottish and English sites, social workers will continue to assess the family, help to engage them with support/clinical services and have their services scrutinised intermittently in government-mandated inspections. In addition, during the trial, there will be regular discussions about service delivery with all local authority colleagues in regular local study Steering Groups and liaison with professionals from other key services relevant to children’s care placements [33].

We anticipate that the nature and intensity of services as usual will vary across different geographical areas and across time during the trial. The Glasgow version of SAU—run by the Family Assessment and Contact Service (FACS)—is an ideal attention control for NIM because it also offers a relationship-based assessment of the parental capacity to care for the child, but has a social work ethos, including more naturalistic observations of the family and unstructured assessments of case files. It does not contain an infant mental health treatment component. In Renfrewshire, the Family Assessment and Contact Team (FACT) offers an assessment using a similar framework. Current SAU in the relevant London boroughs are relatively sophisticated compared to many areas of the UK because of their close relationship to South London and Maudsley Trust (SLaM) which is a partner in this collaboration. For example, state-of-the-art interventions for foster carers such as Dozier’s Attachment and Biobehavioural Catch-up (http://abcintervention.com/) are available for some families. The London Boroughs involved in the trial adopt one of three different methods for assessing families. The first is an assessment delivered by a specialist team within the borough who will offer an assessment of parenting capacity (as with the Glasgow and Renfrewshire models). The second is an assessment carried out by the allocated social worker with additional services (e.g. child and adolescent mental health services) engaged if the social worker deems this to be required. The third is a specialist assessment carried out by an independent social worker/psychologist/psychiatrist. All three methods of assessment must be undertaken within 26 weeks as per the expectations set out by the Judiciary in London. Further exploration of the detailed nature of SAU in London is a key objective of qualitative mapping and modelling we are currently undertaking and is included as part of the trial process evaluation. The heterogeneous nature of services as usual across the Local Authorities feeding into the London and Glasgow sites is seen as a strength in that it will allow for a more detailed exploration of what works, for whom and why. In contrast with NIM, the systems of care that comprise SAU have developed iteratively, borne out of local authority contexts and processes (constrained by local statutory guidance), and have never been formally evaluated.

### Intervention description {11a}

The experimental intervention is NIM, which aims to improve the mental health of young, maltreated children. It has a US evidence-base, although not derived from an RCT [34]. NIM is an intensive, targeted, individualised, family-based intervention that aims to offer assessment and trial-of-treatment for the birth family and make timely recommendations for rehabilitation back to the birth home or adoption.

We have found only one intervention, NIM, that uses an infant mental health approach aiming to improve the quality of permanent placement decisions so that children can experience appropriate nurturing care as early in life as possible [34]. The Tulane Infant Team, who developed NIM, assesses the mental health and relationship quality of every maltreated child under 5 years of age on reception into care. A tailored intervention is then offered to each family aiming to improve parent-child relationships and child mental health. These assessments and the degree of change achieved through intervention inform recommendations to the legal system about the permanent future care of the child. Where significant change has been achieved, it is recommended that children are rehabilitated back to the birth family. If not, the recommendation is adoption. An evaluation of the 4 years prior to, compared with the 4 years after, the introduction of the NIM in the US suggested that the programme effects an increased rate of adoption and, for those returned to birth families, a risk reduction of more than 50% in repeated maltreatment for both that child and subsequent siblings [34]. A follow-up of children several years after exposure to NIM in infancy has shown that on many mental health measures graduates of NIM, whether adopted or rehabilitated to birth families, differed only slightly from the general population [35]. These are apparently remarkable findings, when the high rates of psychopathology in populations of children in care are considered [36] but should be viewed with caution because these studies did not involve randomisation or control interventions and it is possible that other factors contributed to these outcomes. In particular, the Adoption and Safe Families Act, which placed tighter legal guidelines around judicial decision-making about young children in care, came into force just after the NIM service started in Louisiana [37] and it is quite possible that this could account for the improvement in children’s outcomes. In addition, the extent to which these findings could be generalised to a UK context is not known: in Louisiana, there is very little child welfare social work offered to families in the community [32] so NIM has never been tested against the Case Management that is routinely offered as part of UK social care.

The NIM intervention is as follows: a multidisciplinary infant mental health team comprising psychologists, a psychiatrist, other therapists and social workers assesses the mental health and relationship quality of children under 5 years of age upon reception into care. The assessment, which involves each actual and potential caregiver, is manualised and standardised and uses structured interviews, self-report measures and observations. A multidisciplinary meeting is then held to decide if it is appropriate to proceed to a tailored trial-of-treatment. Wherever possible, treatment is offered to each family, drawing on a small range of relationship-based therapeutic techniques all of which comply with the recommendations of a meta-analysis that examined ways of improving parental sensitivity [38]. Parents are also referred as required to other agencies for help with substance misuse, mental health issues or intra-familial violence. These assessments, and the degree of change achieved during the trial-of-treatment, inform recommendations to the legal system about the permanent future care of the child. The aim is to have “the best outcome possible for [the] particular child” (Zeanah, personal communication, 2014), be this a recommendation of rehabilitation to birth family or adoption [34]. Where significant change has been achieved, children are rehabilitated back to the birth family. If not, the recommendation is adoption. Throughout the time that birth families are receiving the intensive intervention, the child remains in a nurturing placement with foster carers who are ideally willing to adopt the child if necessary, although the feasibility trial has shown that foster carers being dually registered as adopters is rare in the UK [39].

In this definitive trial, NIM will be delivered by the Glasgow Infant and Family Team (GIFT) and the London Infant Family Team (LIFT).

**Fig. 1:**
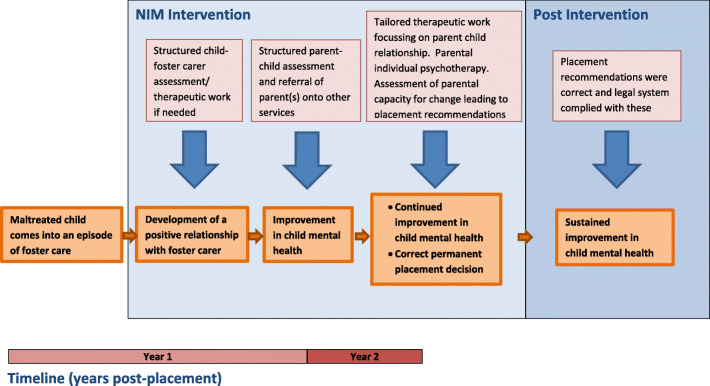
The logic model, NIM

A logic model for NIM (Fig. 1) was developed in collaboration with the developers (Professors Charley Zeanah and Julie Larrieu) prior to the feasibility RCT and may be modified, as a result of process evaluation findings, prior to any roll-out of NIM should it be proven cost-effective.

### Modifications

Two modifications of NIM have become necessary for the definitive trial.

#### Modification to be used in responding to unanticipated service demands

During the period of randomisation-before-consent, the NIM team had significant problems with capacity. This happened again after the first COVID lockdown when social services were cut back. We therefore consulted with the Tulane team for advice on how they had coped with surges in demand. One such surge in demand occurred after Hurricane Katrina, in 2005, when the Tulane Infant Team experienced a 50% increase in referrals. Their advice, based on this experience, was to offer more limited infant mental health input to the entire target population wherever possible. With their guidance (sought by email and phone, and captured for the process evaluation in a focus group in January 2016), the NIM team developed what was termed “NIM light”, for use only during situations of sudden increase in demand. The components of NIM light were:

- Infant mental health-focussed consultation to social work Case Managers
- And/or strategic consultation with service managers to prioritise where NIM can have the most impact for those children on any waiting list—again with an infant mental health lens
- And/or provision of NIM assessment (possibly truncated, e.g. if lack of engagement) without provision of trial-of-treatment (e.g. if the family had demonstrated by non-attendance that unlikely to be motivated towards treatment)
- A further modification was to use an abbreviated assessment, e.g. with abbreviated WMCI and Crowell

Since the study is using an intention-to-treat approach to analysis, we will carefully log those families who received NIM light, rather than NIM (including the amount of NIM they received) as well as any families who, for whatever reason, were randomised to NIM but did not receive the intervention at all.

An additional UK-specific challenge is that routine infant mental health services for babies and pre-school children do not exist in the UK so that, if the NIM teams identify mental health problems in children involved in their trial-of-treatment, there may be no relevant service to refer the child and family on to, which has contributed to slow throughput through NIM. We have addressed this, in Glasgow, by instituting a small multi-agency group called the “Traffic Light Operational Group” that reviews progress through the GIFT team using a traffic-light system which flags families as “red”, “amber” or “green” according to their progress through key points in the NIM timeline. In London, this role is taken by the Judiciary who mandate timescales by which time LIFT must report to them. Should the process evaluation suggest it, we may recommend that NIM light, the TLOG and/or Judicial mandating of timelines become part of the service specification for any future roll-out of NIM in the UK social care and health systems.

#### Modification for the COVID-19 pandemic

Although parenting capacity assessments (by either NIM or services as usual) were deemed to be essential services during the COVID-19 pandemic, referrals of new cases to both arms of the trial had to cease between March and July 2020 because national lockdown restrictions precluded birth families from having contact with their children. Since the legal systems in both England and Scotland regarded observation of live contact between parents and children to be a crucial part of judicial decision-making about children’s care placements, little intervention from either NIM or SAU was possible during these dates. Gradually, each local authority in the study, as well as the NSPCC, developed safe systems for birth families to meet their children and have these contacts observed. Necessary modifications included workers being designated as “essential workers”, availability of Personal Protective Equipment, availability of rooms large enough for safe social distancing to take place and additional cleaning of premises. In tandem, there was rapid development of systems for some contacts and for the standardised assessments and treatments offered as part of NIM to be delivered remotely. Since April 2020, both NIM and services as usual have offered a mix of face-to-face and remote assessments. The challenges involved in service delivery during the pandemic, and the quality of this service delivery, are being examined as part of the process and health economic evaluations. Sensitivity analyses will be conducted to examine any quantitative impact on trial outcomes.

## Criteria for discontinuing or modifying allocated interventions {11b}

If there is a sudden surge in demand for NIM, then, if multi-agency partners agree, NIM light may be offered (see above) to reduce the waiting list and ensure that all of the target population are offered a service.

### Strategies to improve adherence to interventions {11c}

To optimise adherence to NIM, both the GIFT and LIFT teams were trained directly by members of the Tulane Infant Team. GIFT was offered weekly video-conferencing with Charley Zeanah and/or Julie Larrieu for the first 2 years they were operational. Once the LIFT team were operational, they received regular video-conferencing supervision from both the Tulane Infant Team and the GIFT team who, by this time, were expert in delivering NIM. During the feasibility trial, Charley Zeanah and Julie Larrieu scrutinised 18 anonymised and randomly selected reports from the GIFT team with a view to instituting any necessary quality improvements and this will continue during the definitive trial. In addition, in September 2018, we held an away day with members of the Tulane Infant Mental Health Team, GIFT and LIFT to establish, together, what the core components of NIM are—and this was recorded as a focus group to be reported as part of the process evaluation.

To maximise families’ adherence to NIM, families are asked to sign a service agreement prior to beginning the intervention and the importance of attendance for reporting to the legal system is emphasised. Similar processes are conducted for services as usual although these are more formal in England (where social workers are formally tasked to report back to the court by a certain date) than in Scotland where reporting requirements to the Children’s Hearing System are more variable.

### Relevant concomitant care permitted or prohibited during the trial {11d}

There are no restrictions on concomitant care.

### Provisions for post-trial care {30}

There are no provisions for post-trial care other than services to which the NIM and SAU workers decide to refer families.

### Outcomes {12}

The primary outcome is child mental health at 2.5 years post-randomisation (see below).

Secondary outcomes, at 2.5 years post-randomisation, are:

- Time taken to reach a permanent placement (TTPP) (be that adoption or return to birth family without social work supervision)
- Parent/carer-child relationship quality
- Cost-effectiveness

#### Primary outcome measure

Our primary outcome is child mental health, and our primary outcome measure is the Strengths and Difficulties Questionnaire (SDQ). This is a brief behavioural screening questionnaire for 2–16-year-olds (therefore always appropriate at time 3 even if the child entered the study near birth), completed by the primary caregiver (i.e. the caregiver with whom the child is living), with 25 items in 5 subscales: emotional symptoms, conduct problems, hyperactivity/inattention, peer relationship problems and prosocial behaviour [40]. It is sensitive to change in intervention studies, and effect sizes are moderate to large [41–43]. Our review of the literature suggests that SDQ is the most widely used and well-validated measure of mental health in children.

#### Secondary outcome measures

These address the other key components of our NIM logic model (Fig. 1).

1. Parent- or carer-child relationship. This will be measured using the Parent-Infant Global Assessment Scale (PIR-GAS) [44].
2. Time between first care episode and permanent placement decision (adoption or rehabilitation). This will be determined through scrutiny of routinely held social work data.

There is no risk of unblinding with either of these secondary outcome measures as they are rated/entered independently from the research team.

#### Other outcome measures

The range of measures we have included reflect the fact that mental health in infancy is multi-faceted and the various aspects (relationship, psychiatric diagnoses, cognition and language) overlap with one another [45].

The Infant-Toddler Social-Emotional Assessment (ITSEA) is a well-validated parent/carer-completed questionnaire covering a wide range of social and emotional behaviours in infants and pre-school children [46]. It has been shown to be sensitive to change in previous intervention research with maltreated children with medium to large effect sizes [47] and has good longitudinal stability.

Because mental health in pre-school children is so linked to cognitive functioning, we have included a full-scale IQ measure (see Table 2), WPPSI [48], measured at age 2.5 years. WPPSI is a well-validated and commonly used measure for this age group and covers both performance and language aspects of cognition.

**Table 2:** Study procedures

Study procedure	Visit 1	Visit 2	Randomisation automated system	Visit 3 (baseline) ***4–14 weeks post-care entry***	Visit 4 (follow-up) ***15 months post-care entry***	Visit 5 (final follow-up) ***2.5 years post-randomisation***	Interview or focus group
Provision of study information	✓	✓					
Consent		✓					
Randomisation			✓				
SDQ *To be completed for children aged 2 and over, according to age of child:* *• 2*–*4y, 4–17y*				✓	✓	✓	
PIR-GAS *To be completed for all children*				✓	✓	✓	
ITSEA *To be completed for children aged 12–35 months*				✓	✓		
DAI *To be completed if child 12 months and over*				*✓*	*✓*	*✓*	
DAWBA *To be completed for children aged 2 and over, according to age of child:* *• 2*–*4y, 4–17y*				✓	✓	✓	
Service use questionnaire *To be completed for all children*				✓	✓	✓	
TIMB *To be completed for all children*				✓	✓	✓	
Observational Checklist for RAD *To be completed if child 12 months and over*				✓	✓(if not collected V3)		
Cognitive assessment • *WPPSI III—30–47 months,* • *WPPSI III—48–87 months* • *WISC IV—children + 87 months*				✓ (optional)	✓ (optional)	✓	
PedsQL *To be completed for all children, according to age of child:* • *1–12 m, 13*–*24 m, 2–4y, 5–7y*				✓	✓	✓	
RPQ *To be completed if child 12 months and over*				✓	✓	✓	
Data linkage						✓	
Parent/carer qualitative interview/focus group							✓

Psychiatric diagnoses will be assessed using the Development and Wellbeing Assessment (DAWBA)—a validated semi-structured interview generating ICD and DSM diagnoses [49].

The Relationship Problems Questionnaire [50] and the Disturbances of Attachment Interview (DAI) [51] will be used to investigate Reactive Attachment Disorder (RAD) symptoms. The Observational checklist for RAD [52] will be used, alongside these measures, to establish diagnoses of RAD.

Because the commitment of carers to their child has been shown to be related to the quality of the relationship [53], we will use the This is My Baby (TIMB) questionnaire—a brief questionnaire investigating carers’ long-term view of their relationship with the child [54].

In order to adhere to the recommended methods for economic evaluation of public health interventions by the National Institute for Health and Clinical Excellence (NICE) [55], the PedsQL, a validated measure of child Quality of Life [56], will be included with the intent to map outcomes to EQ-5D utility so as to estimate quality-adjusted life years (QALYs). Recent work by Khan et al. [57] has mapped EQ-5D utility scores from the PedsQL generic core scales; hence, these algorithms will provide an empirical basis for estimating health utilities in this population.

Linkage with routine data will allow us to measure repeat episodes of maltreatment and validated physical and mental health diagnoses. Consent for this is sought at recruitment.

Our assessment of effectiveness and cost-effectiveness, based on our primary outcome (mental health), will be measured at 2.5 years after randomisation. We have chosen this time point because, by then, the great majority of children should be settled in a permanent placement (either in a rehabilitated birth family or in an adoptive family). While even the children who were youngest at entry to care will, by this time, be of an age where a valid psychiatric and cognitive assessment can be made using measures that could be repeated at older ages if funding were found to continue following the cohort after this study is complete.

### Participant timeline {13}

Study procedures are summarised in Table 2.

### Sample size {14}

Initially, the target sample size was 492 families to achieve 90% power with a loss to follow-up of 75% [25]. Although 80% power is usual, the Glasgow clinical trials unit routinely powers trials at 90%, especially for complex studies such as this where allowance is needed to be made for unexpected loss to follow-up and missing data. However, the feasibility trial showed that 2.5-year follow-up was > 70% so, when varying the contract with the funder, a revision was made such that our target is now 80% power. Our estimated sample size to achieve 80% power with a loss to follow-up of 30% is 396 families.

### Recruitment {15}

This study uses the novel recruitment and retention strategy of employing experienced social workers, who receive additional specific training in Good Clinical Practice, to screen for eligibility and conduct our study information and consent meetings with potential participants. This is important for several reasons: (1) Social workers are already well trained in engaging with families undergoing crises, in evaluating whether a potential participant is competent to give consent and, if not, whether there are supports that can be put in place to ensure competence (e.g. the presence of an advocate, returning once the participant has had time to discuss the study with a trusted person). (2) Experienced social workers are confident in approaching “gatekeepers”, such as other social workers or their managers, to explain the study and facilitate the opportunity for potential participants to be informed. (3) Social workers have access to data systems that can give crucial information about the whereabouts and life circumstances of potential participants to ensure that it is ethical and safe to conduct an information meeting, or to approach a participant for follow-up.

In addition, in each site, efforts have been made to build good relationships with the most senior social worker in charge of child protection and to involve that person in the local steering group. This is important because these senior managers can mandate that every eligible family in their area should be informed about the study; they can trouble-shoot challenges regarding recruitment and retention with colleagues in other areas during steering group meetings; and they can authorise the research team having access to social work data systems.

## Assignment of interventions: allocation

### Sequence generation {16a}

Random allocation of families will be performed using a mixed minimisation/randomisation method, stratified within the study site. A randomisation schedule will be prepared for each site, in blocks of 10: in each block, 8 allocations will be decided by minimisation, and two at random (one to each group). For those to be minimised, the schedule will indicate which group to allocate to in the case of “no preference” according to the algorithm (4 to each group, at random).

Minimisation is designed to ensure balance of allocations with respect to study site, the age of the youngest child coming into care at the point of randomisation (< 2/≥2 years), the number of children coming into care at the point of randomisation (1/> 1), whether the birth family is fluent in English and the type of care (i.e. foster or kinship)

### Concealment mechanism {16b}

Randomisation is conducted via a Web portal, which requires a log in and password, and is managed by the Robertson Centre for Biostatistics, University of Glasgow. Access rights are allocated to relevant staff, as listed on the trial delegation log, in advance of the randomisation procedure.

### Implementation {16c}

Participants are enrolled on the randomisation system by recruitment coordinators. Recruitment coordinators, trial managers and nominated contacts per site and per arms of trial are automatically informed of the intervention allocation by email. Recruitment coordinators inform the social worker managing the families’ case of arm of trial, and the social worker will then work with the family to manage access to the arm of trial.

## Assignment of interventions: blinding

### Who will be blinded {17a}

The study is single blind. Participants will be aware to which arm of trial they have been allocated. Researchers and statisticians will not. For questionnaire and interview measures, we have incorporated a system whereby the research assistants have a script that they use as families are invited to assessments that aims to ensure they do not reveal intervention allocation (e.g. by stating the location at which parenting capacity assessments were conducted). Following assessment visits, research assistants state which intervention they think the family were attending so that, at the end of the study, we can assess the degree to which these blinding measures were successful. Parenting quality assessments (PIR-GAS) and Time to Permanent Placement are fully blinded since PIR-GAS is rated by individuals who have no access to group allocation and TTPP through examination of social work data, again by individuals with no access to group allocation.

### Procedure for unblinding if needed {17b}

Unblinding is permitted by the independent members of the Data Monitoring and Ethics Committee (DMEC) and, should they wish to become unblinded, these data will only be discussed during closed meetings of the DMEC.

## Data collection and management

### Plans for assessment and collection of outcomes {18a}

Standard operating procedures will be produced for the collection of each measure, and these will be included in training documentation and the study manual. All research nurses or psychologists will be trained in administering study measures and will practice administration of measures with each other prior to conducting research assessments.

All data will be collected using standardised paper case report forms (CRF) based on the individual instruments being used. These will be version controlled.

For the measures requiring rating, more extensive training will be offered until raters achieve sufficient reliability. Once trained to reliability, inter-rater reliability will be regularly checked according to a standard operating procedure in which 10–20% of tests will be randomly selected for independent rating by a second rater. Any difficult ratings, or ratings for which independent raters do not sufficiently agree, will be brought to an approximately 3-monthly conference with an expert rater. The proportion of ratings requiring conferencing will be recorded. Data collection forms are available on request.

### Plans to promote participant retention and complete follow-up {18b}

Because the study population are families with a wide range of social and psychological difficulties, it will be essential for the research team to have access to social work datasets for tracking purposes and to employ experienced social workers to re-engage with families at follow-up. All attempts will be made to gather data from consented families, including those who have failed to adhere to intervention protocols. Repeated attempts to contact participants will be made by phone and, later, in “drop-in” visits—each time leaving instructions regarding how to text or phone the team if the participant no longer wishes to participate in that assessment. Unless participants have stated that they do not give consent, routine data (e.g. for Time to Permanent Placement) will be gathered, even if participants do not respond to attempts to contact them.

### Data management {19}

All data handling procedures will be detailed in a study-specific data management plan.

All data collected will be stored securely, in accordance with the University of Glasgow Best Research Practice Guidelines, and managed in accordance with the Data Protection Act 1998 and the General Data Protection Regulation (from May 2018), in either locked filing cabinets or password-protected databases. Data will be accessible only by members of the UoG research team and their research partner KCL. All quantitative data collected as part of the study will be securely transferred to the Robertson Centre for Biostatistics CTU for data entry and checking in accordance with their SOPs. Qualitative interview data will be transcribed by an external transcriber and securely stored.

Data will be validated at regular intervals during the study. Data discrepancies will be flagged to the study site, and any data changes will be recorded to maintain a complete audit trail (reason for change, date change made, who made change).

### Confidentiality {27}

All data collected will be kept separate from any individual participant identifiers and secure. Participants will be assigned a unique ID number to link their data throughout the trial.

### Plans for collection, laboratory evaluation and storage of biological specimens for genetic or molecular analysis in this trial/future use {33}

No biological specimens will be collected.

## Statistical methods

### Statistical methods for primary and secondary outcomes {20a}

The primary analysis will use a generalised linear mixed effects regression model for the primary outcome measure to account for clustering of outcomes within families and adjusting for child age and other minimisation factors. The primary outcome measure is the Total Difficulties scale of the SDQ at 2.5 years after entering care. All models will be adjusted for age at the point of completion.

A single model over all three time points will be fitted. Interaction terms will be used to assess whether intervention effects vary between subgroups. The level of significance for the primary outcome is 0.05. A detailed Statistical Analysis Plan will be prepared and approved prior to database lock and unblinding of intervention groups.

We are analysing the trial on an intention-to-treat basis. Families who re-enter the study will not be re-randomised and, if appropriate, will receive assessment and treatment by the team to which they were previously randomised.

Blinded data analyses will be carried out during the trial, to determine the most appropriate method of allowing for clustering of outcomes within families. All modelling decisions taken during blinded analyses will be documented and approved prior to database lock. If modifications to the analysis are required following database lock, these will be documented and justified within the final statistical results.

### Interim analyses {21b}

No interim analyses are planned, other than blinded analyses to determine whether data need to be transformed prior to analysis, or if an alternative link or variance functions are required within the model. These modelling decisions will be documented and approved prior to database lock. While we do not consider it necessary to pre-specify stopping rules, as the feasibility trial has not suggested any harm, data monitoring is the responsibility of the Data Monitoring and Ethics Committee which can examine unblinded data and recommend stopping the trial if thought necessary at any point during the study.

### Methods for additional analyses (e.g. subgroup analyses) {20b}

The following subgroups will be considered:

- Study site
- Deprivation, as determined by (Scottish) Index of Multiple Deprivation quintiles
- Age of youngest eligible child coming into care at the point of randomisation (< 2/≥2 years)
- Age of individual children (< 2/≥2 years)
- Number of eligible children coming into care at the point of randomisation (one/more than one child)
- Birth family fluency in English
- Sex
- Randomisation system in place at the time of randomisation

The primary and secondary outcomes will be summarised overall and by randomised group, separately within each subgroup, and the primary and secondary analysis regression models will be extended to include intervention-by-subgroup interaction terms, to estimate intervention effects within subgroups, and to test for heterogeneity in intervention effects between subgroups.

### Methods in analysis to handle protocol non-adherence and any statistical methods to handle missing data {20c}

Analyses will be according to the intention to treat principle, in that they will be in relation to randomised allocation, regardless of compliance with the allocated intervention. As a pragmatic trial, it is unlikely that any protocol deviations will necessitate removal of any participants from analysis. However, protocol non-adherences will be logged with the clinical trial unit and will be assessed for their impact on the scientific integrity of the trial.

Missing outcome data will not be imputed in the main analyses, though the primary analysis is based on a linear mixed effects regression model which accounts for missing data, and sensitivity analyses will be carried out to (a) assess alternative assumptions regarding missing outcome data and (b) use multiple imputation methods, assuming data are missing at random from questionnaires.

### Plans to give access to the full protocol, participant-level data and statistical code {31c}

When the trial is completed, requests can be made for an anonymised version of the dataset.

## Oversight and monitoring

### Composition of the coordinating centre and trial steering committee {5d}

The study coordinating centre is the Glasgow Clinical Trials Unit (CTU Registration Number 16). Day-to-day trial coordination is conducted by the chief investigator and senior trial manager, with oversight of the entire study, and the English site is coordinated by a London principal investigator and London trial manager. The entire Glasgow and London research team (both trial managers, chief and principal investigator, Glasgow and London recruitment coordinators and Glasgow and London research nurses) will meet approximately monthly during the most intense periods of recruitment and retention and less frequently during less intense periods of the study. The process evaluation team (Glasgow based) will meet approximately every 6 weeks and there will be frequent (at least weekly) ad hoc meetings between trial managers and various members of the study team. The trial management group (comprising the co-investigators) will meet approximately quarterly, with two of the meetings each year timed to just precede the six-monthly data monitoring and ethics committee meetings which will precede the six-monthly trial steering committee meetings. Additional DMEC and TSC meetings can be convened on an ad hoc basis if there are specific urgent issues to discuss.

### Composition of the data monitoring committee, its role and reporting structure {21a}

A Data Monitoring and Ethics Committee will act as the oversight body for this trial with its primary roles being to safeguard the interests of trial participants, monitor the main outcome measures including safety and efficacy, and monitor the overall conduct of the trial.

The committee compromises 3 independent members, one of whom is the chair. Independent members are nominated to and approved by the funder. Members are chosen for their expertise in conducting relevant research. The independent members include a statistician and a psychiatrist. The committee facilitator is the trial manager. Other attendees include the chief investigator and trial statistician. The committee will meet at least annually and reports to the funder. The charter is available upon request.

### Adverse event reporting and harms {22}

To ensure that serious adverse events relevant to the trial are captured, the study team will review participants’ case notes on a six-monthly basis for the presence of serious adverse events. Deaths as recorded in routinely available health data in Scotland will also be examined. All SAEs arising during the trial will be recorded as soon as reasonably practicable after the site first becomes aware of the event.

Many participants and their birth parents taking part in the trial will have complex medical and mental health histories. Therefore, many adverse events and serious adverse events would be expected to occur within the participant group irrespective of their involvement within the study. These expected events do not necessarily require reporting to the Research Ethics Committee as serious adverse events but will be monitored by the trial team in relation to differential rates across trial arms, via the usual health and social care processes to ensure participant safety, and details recorded in a log. Their relatedness will be assessed by the chief investigator and, 6 monthly, by a small panel of experts (including a consultant paediatrician). The Data Monitoring and Ethics Committee (DMEC) will review these six-monthly.

Any SAE occurring to a research participant (or, in the case of deaths, also, their birth parent) where in the opinion of the chief investigator (CI), the panel of experts or the DMEC, the event was “Related”—that is, it resulted from the administration of any of the research or intervention procedures, and “Unexpected”—that is, the type of event is not an expected occurrence, will be reported by the Pharmacovigilance office to the Research Ethics Committee that approved the trial.

### Frequency and plans for auditing trial conduct {23}

The trial is sponsored by NHS Greater Glasgow and Clyde and subject to the protocols in place for monitoring and audit. These are independent of trial conduct. The trial was audited in September 2016.

### Plans for communicating important protocol amendments to relevant parties (e.g. trial participants, ethical committees) {25}

Any important protocol modifications will always be developed with the TMG, reported to the funder and will be discussed by the DMEC and discussed/ratified by the TSC. The protocol will be updated in the trial registry. The local steering groups will be the main method for communicating any important protocol modifications to relevant parties involved in supporting recruitment to the study.

Any deviations from the protocol will be fully documented using a breach report form.

## Dissemination plans {31a}

A publication plan is reviewed at each Trial Steering Committee and all submitted publications are logged with the funder. In addition, a dissemination plan will be co-created with the User-Professional Group to ensure results are made available accessibly.

## Discussion

Although a short version of this protocol was published in 2016, we wished to publish a longer version because we have been required to modify aspects of the protocol subsequently in response to judicial concerns and the need for amendments to timescales and recruitment targets. In addition, certain aspects of the study methodology are novel. First, the role of infant mental health assessment and treatment has never previously been rigorously examined in the context of judicial decisions about children in the care system. Second, this is the first modern randomised controlled trial conducted in the context of the UK family courts [58]. Randomised controlled trials have only rarely been conducted in legal contexts internationally [59], and it has been challenging to square medical routes to gathering evidence (which have been trial-based for more than 80 years) with legal routes to gathering evidence which has been precedent based [59]. Third, this is the first trial that we are aware of that aims to recruit families who have recently had their children taken into care. As we have described previously, this makes informed consent challenging and we aim to learn from good practice in previous trials, e.g. those conducted with patients after head injury [60], to ensure we adhere to good clinical practice [26]. Fourth, the monitoring of serious adverse events has never been conducted in this context and we aim to use innovative methods to both detect and evaluate potential SAEs.

These specific challenges will necessitate a careful focus on ethical principles in trials. In particular, the oversight by senior practitioners of each participating family’s eligibility for, and ongoing participation in, the study will require regular review both in multi-agency steering committees and through the development of excellent relationships between these practitioners and the trial team.

## Trial status

Recruitment to the feasibility trial begun in January 2012 and was ongoing at the time of submission. Recruitment ended on 31 July 202. The trial was operating under version 7.0 (12.05.2020) of its approved protocol at the time of submission.

## Abbreviations

ADHD: Attention deficit and hyperactivity disorder
BeST: Best Services Trial
CI: Chief investigator
CM: Case Management
CRF: Case report form
CSO: Chief Scientists Office
CTU: Clinical Trials Unit
DAI: Disturbances of Attachment Interview
DAWBA: Development and Wellbeing Assessment
DHSC: Department of Health and Social Care
ESS: Emotional Signalling Scale
DMEC: Data Monitoring and Ethics Committee
GIFT: Glasgow Infant and Family Team
ITSEA: Infant and Toddler Social and Emotional Assessment
KCL: King’s College London
LIFT: London Infant and Family Team
NHS: National Health Service
NHS GGC: National Health Service Greater Glasgow and Clyde
NIHR: National Institute for Health Research
NIM: New Orleans Intervention Model
NSPCC: National Society for the Prevention of Cruelty to Children
PedsQL: Paediatric Quality of Life
PIR-GAS: Parent and Infant Relationship Global Assessment Scale
QALY: Quality-adjusted life years
RCB: Robertson Centre for Biostatistics
RCT: Randomised controlled trial
REC: Research Ethics Committee
RPQ: Relationships Problems Questionnaire
SAE: Serious adverse event
SAU: Services as usual
SDQ: Strengths and Difficulties Questionnaire
SOP: Standardised Operating Procedure
TIMB: This Is My Baby
TMG: Trial Management Group
TSC: Trial Steering Committee
TTPP: Time To Permanent Placement
UK: United Kingdom
UoG: University of Glasgow
UPG: User Professional Group
US: United States
WPPSI: Wechsler Pre-school and Primary Scale of Intelligence
